# The value of cryoablation in orbital surgery

**DOI:** 10.1007/s00508-024-02340-6

**Published:** 2024-03-14

**Authors:** Johannes Herta, Christian Matula, Lisa Wadiura, Matthias Millesi

**Affiliations:** Department of Neurosurgery, Medical University of Vienna, Währinger Gürtel 18–20, 1090 Vienna, Austria

**Keywords:** Cryosurgery, Orbital tumors, Cryoadhesion, Orbital schwannoma, Cavernous venous malformation

## Abstract

Aim of this article is to give an overview of the technical background and the advantages of modern devices for different applications of cryoablation in cranio-orbital neurosurgery.

The treatment of orbital lesions is complicated by the complex and potentially inapparent anatomy due to retro-orbital fat. With the help of cryoprobes different well-defined lesions such as cavernous venous malformations can be safely and effectively removed thanks to the cryoadhesive effect. Their use has been described in several different approaches including traditional lateral or transcranial orbitotomy but also anterior transconjunctival as well as transnasal endoscopic approaches. Recently, single-use devices were introduced that allow the use of cryosurgery also without the need for large investment or service costs.

**Supplementary Information:**

The online version of this article (10.1007/s00508-024-02340-6) contains supplementary material, which is available to authorized users.

## Introduction

In orbital tumors treatment is typically indicated in growing lesions or if they become symptomatic [1, 2]. Such typical symptoms include orbital protrusion, double vision, and visual decline [1, 2]. In most of these cases, complete surgical removal is the treatment of choice. If possible, entire removal of the orbital mass or tumor should be performed without penetration of the lesion [1, 2]. A variety of different surgical corridors are nowadays available including traditional open microsurgical transcranial and transorbital but also endoscopic transcranial, transorbital, and transnasal approaches depending on the different locations of tumors within the orbit [1–8].

Independent of the chosen surgical approach, meticulous preparation and dissection of the orbital mass or tumor without harming surrounding healthy structures such as the globe, ocular muscles or nerves needs to be performed. Eventually, after the lesion is freed from all adhesions, one piece removal should be performed. Different techniques and instruments such as grasping forceps or placement of a suture through the mass can be used; however, the frailty of some of these masses or their capsule puts them at risk of rupture and opening during this maneuver which might complicate lesion removal due to the narrow and complex retro-orbital conditions.

Another option to gently grasp a lesion without perforating it may be to use a cryoprobe [1, 7, 9]. In general, cryosurgery is used in various disciplines for different diseases. These include dermatological surgery, oral and maxillofacial surgery but also general and gynecological surgical applications for tumors and other conditions [10–14]. In most of these treatments the aim is maximum cellular destruction by rapid freezing [15].

Already in 1975 the use of the adhesive effect of a cryoprobe for grasping and gentle removal of orbital lesions was described by Putterman and Goldberg in a small series of 4 patients [9]. Following this, various probes have been developed and used to facilitate orbital tumor removal. Aim of this report is to give an overview of the technical background and different uses together with a discussion of the most appropriate conditions to apply such a cryoprobe in orbital surgery.

## Technical background of cryosurgery

The method of destroying tissue by freezing is called cryoablation. Cryoablation was first introduced by the English physician James Arnott (1797–1883) who used a combination of salt and crushed ice to treat breast, uterine and skin cancer [10, 12]. Since then, it has found many suitable areas of application as it is simple, cheap and safe to use in medicine. Resulting from the testing of various different cooling agents, liquid nitrogen has emerged as the most popular cryogen for medical use with achievable low temperatures of −197 °C but other possible agents used today include noble gases like argon but also oxygen, nitrous oxide or carbon dioxide [14, 16, 17].

In general, the lesion must be targeted with the cryo-probe. Once in physical contact, the probe is rapidly cooled down and removes heat from the lesion by conduction. The cryoprobe contains the pressurized gas which flows out through a narrow opening. During this process the gas rapidly expands at room temperature and exhibits the Joule-Thompson cooling effect [16]. This effect leads to rapid cooling of the metallic probe itself which then transfers the freezing effect to the surrounding tissue [16].

The extent of cellular injury may be influenced by the cooling rate, the target temperature, cooling duration and the thawing rate [17]; however, in nearly all tissues cell death is induced by cooling rapidly below −40 °C [15]. Cell death may occur due to direct injury to cells and indirect mechanisms that induce changes to the cellular microenvironment. Such direct damage occurs through ice crystal formation in the extracellular space during the freezing process [16, 17]. Due to the increased osmolality, more intracellular water is withdrawn, which leads to dehydration of the cells. If cooling occurs rapidly, intracellular ice formation leads to physical cellular damage and cell death [16]. Contrary to the freezing process thawing usually occurs in the extracellular space first and leads to water influx into the cells. This eventually can lead to swelling and bursting which also creates direct injury to the cells [16, 17]. In contrast to the freezing process, more damage occurs if thawing happens slowly [17]. Following this, indirect cell damage occurs via apoptosis processes and inflammatory changes in the surrounding damaged tissue and its microenvironment [16].

In addition to these ablative effects, cryoprobes also have an adhesive effect that became of interest as a tool in modern surgery especially for the use during minimally invasive procedures.

### Cryoablation devices

There are currently several providers of cryoablation devices on the market. The main differences between the devices are: (1) the gas used, (2) the controllability of the gas flow, (3) single or multi-use probes and (4) difference in probe shape.

Regarding different probes, their design varies according to their field of use. In this sense, special designs such as slings are used to cover larger areas or balloons in the setting of intravascular applications [18, 19]. Otherwise, most of the cryoprobes have narrow tips for cryoablation.

In orbital surgery, in multi-use devices the probe can be changed but most of them are designed with a long thin tip [2, 4]. Furthermore, most of these tips are round although attempts were made to introduce flat oblique tips to increase the surface that comes in contact with the lesion for a better adhesion effect [2, 4, 20, 21]. Single-use devices are often wider at the grip as they need to give space for the container with the cooling agent and their probes are fixed [20]. These different aspects need to be considered, especially when different surgical approaches are used [2, 4, 7].

**Fig. 1:**
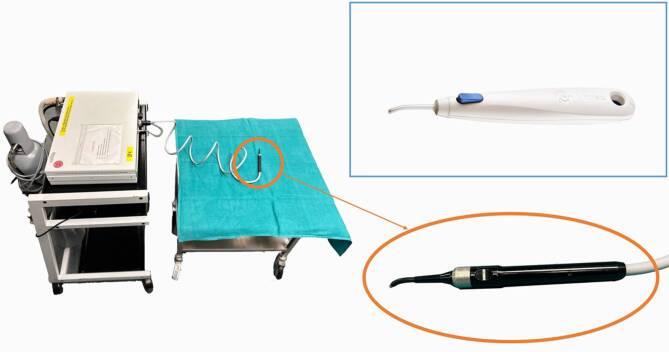
Two different cryoablation devices are shown. The CryoTreq (blue square) is a single-use wireless cryoprobe that integrates its gas container in its handpiece. The Erbecryo (orange circle) on the other hand is a multi-use device that consists of a gas tank, the device itself on a cart and a wired cryoprobe. Different cryoprobes can be used

At our institution we currently use two different cryoablation devices (Fig. 1). CryoTreq (BVImedical, Waltham, MA, USA) is a single-use wireless cryoprobe that has a liquid nitrous oxide container integrated into the handpiece [20]. The clear advantage of this system is that it is an easy to use wireless device with no service or investment costs that can be used up to 1h. The drawback of a single-use device like this is the lack of adjustment options. Additionally, the rear part with the small container is wider and the thin tip shorter and curved in comparison to other devices. In most situations this is sufficient for most of the lesions but to reach to the orbital apex, the superficial opening needs to be large enough. Other single-use devices also exist but are mostly designed for superficial or skin lesions.

On the other hand, the Erbecryo (Erbe Elektromedizin GmbH, Tübingen, Germany) device at our institution needs more space for the equipment cart and the associated carbon dioxide bottle but it allows more flexibility as it can be used with different probe shapes as outlined above. Similar to this, the Optikon Cryoline device (Optikon 2000 S.p.A., Rome, Italy) offered a variety of tips and has been described also in the setting of an endonasal approach to the orbit in the publication by Castelnuovo et al. [4].

Newer cryoablation devices are currently in development. In these devices, rarer noble gases such as argon are used to cool the probe tip by circulation of the cryogen. The aim here is to reduce the costs and size of the devices due to the current loss of gases due to the Joule-Thompson effect [16].

## Discussion

The abovementioned ablative processes of cryotherapy have been used in various surgical disciplines. These included dermatological, oral and maxillofacial, general and gynecological surgery [10–12]. Especially in recent years, the field of cryosurgery received a new wave of interest in the treatment of prostate cancer and breast cancer [13, 14].

### Cryosurgery in surgical procedures of the orbit

Ablative cryosurgery was also used extensively in ophthalmological surgery starting as early as the 1930s with the treatment of retinal tears [10]. To date, cryoablation in ophthalmological surgery remains in use for a variety of different conditions such as glaucoma, cataract, retinopathy, disorders of the eyelid or neoplastic lesion [22–26].

Additionally, the adhesive effect of a cryoprobe came into the focus of interest after the first description of its use for removal of orbital masses [1, 9]. As a result, it became clear that certain advantages exist for both the ablative effect as well as the adhesive effect of cryosurgery during surgery of orbital pathologies. For demonstration of the cryoadhesive effect for removal of a cavernous venous malformation in an illustrative case see video 1 (supplementary material).

### Advantages and disadvantages of cryosurgery

An advantage of cryoablation is the direct visual control of the ablation progress. During cryoablation the lesion undergoes a phase transition forming an ice ball that is attached to the tip of the device. This allows the surgeon to dissect the tumor out of the surgical site very safely and clearly using gentle traction. This means that lesions that are otherwise difficult to grasp can be removed en bloc in a narrow and complex anatomical space due to the intraorbital fat tissue. Another theoretically potential advantage of cooling may be the anesthetic effect that is provided by cryoablation in comparison to resection by thermocoagulation [27].

Furthermore, the developing adhesion through the ice ball formation allows gentle manipulation of an orbital mass and removal with reduced risk of opening the lesion or tearing off a capsule [1, 2]. Moreover, the freezing effect of the tumor itself but also close vessels can lead to reduced bleeding during the removal [1].

### Orbital pathologies suitable for removal with a cryoprobe

After initial case series, the application of the cryoadhesive effect for removal of orbital tumors gained remarkable interest with orbital surgeons [1, 7, 28]. Several studies reported the use of cryoprobes for the removal of different orbital tumors with variable success. The most suitable lesions represent well-circumscribed encapsulated masses with no relevant adhesions or tendency to infiltrate surrounding structures. Therefore, orbital cavernous venous malformations represent the most suitable lesions as they consist of dilated venous channels that are typically surrounded by a thin fragile capsule (video 1, supplementary material) [29, 30]. They are also the most common benign intraorbital mass, typically requiring resection only if they show growth on serial imaging or become symptomatic or lead to disfiguring proptosis [2, 29, 30].

Besides cavernous venous malformations schwannomas, hemangiopericytomas, dermoid or epidermoid cysts and meningiomas within the orbit are also well-circumscribed benign lesions in which the use of a cryoprobe can facilitate complete removal [1, 7, 9]. In addition, some cases of removal of malignant tumors with the use of a cryoprobe were also reported and include rhabdomyosarcoma or carcinomas [1, 7]. In these situations, removal with a cryoprobe can also facilitate en bloc resection without rupture of the lesions and spillage of malignant tumor cells [1, 7].

In pathologies that infiltrate surrounding tissue like the eyeball, muscles but also the surrounding bone, the application of cryosurgery is of limited value [1, 7]. This also applies to diffuse pathologies, although some authors advocate using cryosurgery also for tissue acquisition during biopsy procedures [1, 2, 7].

### Possible approaches for cryoextraction of orbital masses

Traditionally, a lateral orbitotomy (Kroenlein approach) represented the workhorse for the extraction of orbital masses [5, 7, 9, 28]. This applied to both traditional procedures with grasping forceps or threads to resect orbital lesions as well as surgical procedures with the application of a cryoprobe [1, 3, 5, 7, 9]. Over the years, various transcranial approaches but also anterior transconjunctival approaches have been applied and advocated to remove orbital masses with cryoextraction [1–3, 7, 28, 31].

In recent years, the application of a cryoprobe for the removal of intraorbital masses was also used in endoscopic transnasal approaches leading to a small number of published cases reports [4, 6, 8, 32].

## Conclusion

Although cryosurgery has been in medical use for a very long time, it has not yet had a major role in general neurosurgery; However, cryosurgery shows its strength especially when it comes to interventions in the narrow and complex orbital anatomy when removing well-circumscribed lesions. The cryoadhesive effect makes the removal of these lesions gentle and safe. As there are now single-use cryoprobes that do not require a large gas tank, cryosurgical devices should be considered part of every orbital surgeon’s standard armamentarium.

## Supplementary Information

Video 1: Demonstration of cryoextraction in an illustrative case: removal of an orbital cavernous venous malformation via a lateral orbitotomy.

## References

[1] Kiratli H, Bilgiç S. Cryoextraction in the management of orbital tumors. An old technique revisited. Orbit. 1998;17:189–94. 12048727 10.1076/orbi.17.3.189.2753

[2] Millesi M, Pichler L, Denk C, Lukas J, Matula C, Wadiura L. Clinical Outcome and Technical Nuances After Resection of Orbital Cavernous Venous Malformations—A Single-Center Experience. World Neurosurg. 2021;153:e244–9. 34182179 10.1016/j.wneu.2021.06.089

[3] Kannan S, Hasegawa M, Yamada Y, Kawase T, Kato Y. Tumors of the orbit: Case report and review of surgical corridors and current options. Asian J Neurosurg. 2019;14:678–85. 31497084 10.4103/ajns.AJNS_51_19 PMC6703027

[4] Castelnuovo P, Arosio AD, Volpi L, De Maria F, Ravasio A, Donati S, et al. Endoscopic Transnasal Cryo-Assisted Removal of Orbital Cavernous Hemangiomas: Case Report and Technical Hints. World Neurosurg. 2019;126:66–71. 30771539 10.1016/j.wneu.2019.01.235

[5] Dalfino G, Sileo G, Ronchi A, Lazzari E, Castelnuovo P, Turri Zanoni M. Lateral Orbitotomy Cryo-Assisted Removal of Orbital Cavernous Hemangiomas: Case Report and Technical Hints. World Neurosurg. 2023;178:69. 37453728 10.1016/j.wneu.2023.07.023

[6] Leocata A, Veiceschi P, Ferlendis L, Agresta G, Castelnuovo P, Locatelli D. Endoscopic Endonasal Excision of a Cavernous Hemangioma of the Orbital Apex with Cryoprobe Assistance: Two-Dimensional Surgical Video. World Neurosurg. 2023;176:142. 37116786 10.1016/j.wneu.2023.04.088

[7] Rosen N, Priel A, Simon GJB, Rosner M. Cryo-assisted anterior approach for surgery of retroocular orbital tumours avoids the need for lateral or transcranial orbitotomy in most cases. Acta Ophthalmol. 2010;88:675–80. 19732050 10.1111/j.1755-3768.2009.01515.x

[8] Rimmer RA, Graf AE, Fastenberg JH, Bilyk J, Nyquist GG, Rosen MR, et al. Management of Orbital Masses: Outcomes of Endoscopic and Combined Approaches With No Orbital Reconstruction. Allergy Rhinol (providence). 2020;11:215265671989992. 10.1177/2152656719899922 PMC6961138 31984165

[9] Putterman A, Goldberg MF. Retinal Cryoprobe in Orbital Tumor Management. Am J Ophthalmol. 1975;80:88–92. 1155554 10.1016/0002-9394(75)90875-2

[10] Cooper SM, Dawber RPR. The History of Cryosurgery. J R Soc Med. 2001;94:196–201. 11317629 10.1177/014107680109400416 PMC1281398

[11] Clebak KT, Mendez-Miller M, Croad J. Cutaneous Cryosurgery for Common Skin. Conditions. 2020;101. 32227823

[12] Kujan O, Azzeghaiby SN, Tarakji B, Abuderman A, Sakka S. Cryosurgery of the oral and peri-oral region: a literature review of the mechanism, tissue response, and clinical applications. J Invest Clin Dent. 2013;4:71–7. 10.1111/j.2041-1626.2012.00165.x 23001938

[13] Gao L, Yang L, Qian S, Tang Z, Qin F, Wei Q, et al. Cryosurgery would be An Effective Option for Clinically Localized Prostate Cancer: A Meta-analysis and Systematic Review. Sci Rep. 2016;6:27490. 27271239 10.1038/srep27490 PMC4895342

[14] Mokbel K, Kodresko A, Ghazal H, Mokbel R, Trembley J, Jouhara H. The Evolving Role of Cryosurgery in Breast Cancer Management: A Comprehensive Review. Cancers. 2023;15:4272. 37686548 10.3390/cancers15174272 PMC10486449

[15] Baust J, Gage AA, Ma H, Zhang C‑M. Minimally Invasive Cryosurgery—Technological Advances. Cryobiology. 1997;34:373–84. 9200822 10.1006/cryo.1997.2017

[16] Erinjeri JP, Cryoablation CTWI. Mechanism of Action and Devices. J Vasc Interv Radiol. 2010;21:S187–91. 20656228 10.1016/j.jvir.2009.12.403 PMC6661161

[17] Baust JG, Gage AA. The molecular basis of cryosurgery. BJU Int. 2005;95:1187–91. 15892798 10.1111/j.1464-410X.2005.05502.x

[18] Desai V, Sampieri G, Namavarian A, Lee JM. Cryoablation for the treatment of chronic rhinitis: A systematic review. Journal of Otolaryngology—Head & Neck Surgery. 2023;52:s40463-023-00645–6. 10.1186/s40463-023-00645-6 PMC10148426 37120607

[19] Ohkura T, Yamasaki T, Kakita K, Hattori T, Nishimura T, Iwakoshi H, et al. Comparison of maximum-sized visually guided laser balloon and cryoballoon ablation. Heart Vessels. 2023;38:691–8. 36441215 10.1007/s00380-022-02208-7 PMC10085885

[20] Rizzo S, Savastano MC, Gambini G, De Vico U, Caporossi T, Savastano A. New Disposable Cryotherapy Device: A Small, Smart, and Useful Tool. Ophthalmology Retina. 2021;5:598–600. 10.1016/j.oret.2020.10.011 33583563

[21] Finger PT. “Fingertip” cryoprobe assisted orbital tumour extraction. Br J Ophthalmol. 2005;89:777. 15923524 10.1136/bjo.2004.055426 PMC1772669

[22] Andersen C, Phelps D. Peripheral retinal ablation for threshold retinopathy of prematurity in preterm infants. Cochrane Neonatal Group, editor. Cochrane Database of Systematic Reviews [Internet]. 1999 [cited 2023 Nov 18];2010. Available from: 10.1002/14651858.CD001693 10.1002/14651858.CD001693 PMC8406950 10796444

[23] Bartley GB, Bullock JD, Olsen TG, Lutz PD. An Experimental Study to Compare Methods of Eyelash Ablation. Ophthalmology. 1987;94:1286–9. 3684207 10.1016/s0161-6420(87)80013-1

[24] Hajjaj A, Van Overdam KA, Gishti O, Ramdas WD, Kiliç E. Efficacy and safety of current treatment options for peripheral retinal haemangioblastomas: a systematic review. Acta Ophthalmologica [Internet]. 2022 [cited 2023 Nov 18];100. Available from: 10.1111/aos.14865 10.1111/aos.14865 33834636

[25] Yi X, Meng F, Bi Y, He L, Qian J, Xue K. Intraocular medulloepithelioma clinical features and management of 11 cases. Br J Ophthalmol. 2023;bjo-2022-322449. 10.1136/bjo-2022-322449 36997291

[26] Bellows JG. Cryotherapy of Ocular Diseases. Philadelphia: J. B. Lippincott; 1966.

[27] Khezri MB, Akrami A, Majdi M, Gahandideh B, Mohammadi N. Effect of cryotherapy on pain scores and satisfaction levels of patients in cataract surgery under topical anesthesia: a prospective randomized double-blind trial. BMC Res Notes. 2022;15:234. 35765086 10.1186/s13104-022-06125-w PMC9241292

[28] Lazar M, Rosen N, Geyer O, Godel V. A Transconjunctival Cryosurgical Approach For Intraorbital Tumours. Aust N Z J Ophthalmol. 1985;13:417–20. 3833301 10.1111/j.1442-9071.1985.tb00457.x

[29] Calandriello L, Grimaldi G, Petrone G, Rigante M, Petroni S, Riso M, et al. Cavernous venous malformation (cavernous hemangioma) of the orbit: Current concepts and a review of the literature. Surv Ophthalmol. 2017;62:393–403. 28131871 10.1016/j.survophthal.2017.01.004

[30] Rootman DB, Heran MKS, Rootman J, White VA, Luemsamran P, Yucel YH. Cavernous venous malformations of the orbit (so-called cavernous haemangioma): a comprehensive evaluation of their clinical, imaging and histologic nature. Br J Ophthalmol. 2014;98:880–8. 24627253 10.1136/bjophthalmol-2013-304460

[31] Campbell PG, Yadla S, Rosen M, Bilyk JR, Murchison AP, Evans JJ. Endoscopic Transnasal Cryo-Assisted Removal of an Orbital Cavernous Hemangioma: A Technical Note. Minim Invasive Neurosurg. 2011;54:41–3. 21509724 10.1055/s-0030-1270465

[32] Marcellino CR, Peris-Celda M, Link MJ, Stokken JK. Endoscopic Endonasal Resection of Orbital Apex Cavernous Hemangioma: 2‑Dimensional Operative Video. Oper Surg. 2019;16:E144–5. 10.1093/ons/opy192 30085112

